# Curriculum development in Liberia's first postgraduate psychiatry training programme

**DOI:** 10.1192/bji.2024.2

**Published:** 2024-05

**Authors:** Micaela B. Owusu, Temitope Ogundare, Senait Ghebrehiwet, Malveeka Sharma, Miles C. Henderson, Michelle P. Durham, Christina P. C. Borba, Babawale Ojediran, David C. Henderson, Benjamin L. Harris

**Affiliations:** 1 Assistant Professor of Psychiatry, Boston University School of Medicine, Boston, Massachusetts, USA; 2 Psychiatry Resident, Boston Medical Center, Boston University School of Medicine, Boston, Massachusetts, USA Email: ogundare@bu.edu; 3 Program Manager, Global and Local Center for Mental Health Disparities, Department of Psychiatry, Boston Medical Center, Boston University School of Medicine, Boston, Massachusetts, USA; 4 Assistant Professor, Department of Neurology, University of Washington School of Medicine, Seattle, Washington, USA; 5 Research Assistant, Boston University School of Medicine, Boston, Massachusetts, USA; 6 Vice Chair of Education, Department of Psychiatry, Boston University School of Medicine, Boston, Massachusetts, USA; 7 Vice Chair of Research, Department of Psychiatry, Boston University School of Medicine, Boston, Massachusetts, USA; 8 Head of Psychiatry, John F. Kennedy Memorial Medical Center, Monrovia, Liberia; 9 Chair of Psychiatry, Boston University School of Medicine, Boston, Massachusetts, USA; 10 Chair of Psychiatry, A.M. Dogliotti Medical College, University of Liberia, Monrovia, Liberia

**Keywords:** Curriculum development, Liberia, medical education, capacity building, postgraduate training

## Abstract

This paper describes the implementation of curricula for Liberia's first-ever psychiatry training programme in 2019 and the actions of the only two Liberian psychiatrists in the country at the time in developing and executing a first-year postgraduate psychiatry training programme (i.e. residency) with support from international collaborators. It explores cultural differences in training models among collaborators and strategies to synergise them best. It highlights the assessment of trainees’ (residents’) basic knowledge on entry into the programme and how it guided immediate and short-term priority teaching objectives, including integrated training in neuroscience and neurology. The paper describes the strengths and challenges of this approach as well as opportunities for continued growth.

Liberia had no postgraduate (i.e. residency or specialty training) programme in psychiatry until 2019, when the Liberia College of Physicians and Surgeons (LCPS) launched the Liberia Psychiatry Residency Program in partnership with the Department of Psychiatry at Boston Medical Center, USA. A 10-year working relationship between the faculty of the two institutions enabled transatlantic collaboration to train Liberian physicians to become psychiatrists and meet the unique clinical needs of the Liberian population. Liberia was destabilised by two decades of military and political conflicts that disrupted the healthcare system, leaving few mental health services for a severely traumatised population. Although international organisations employed ‘psychosocial agents’ to meet basic mental health needs, the training of these individuals was brief and lacked supervision owing to a dearth of highly trained Liberian mental health professionals.^1^

An article published elsewhere details the steps to developing and launching the Liberia Psychiatry Residency Program.^2^ The curriculum was adapted from international postgraduate core competencies and the West African College of Physicians’ (WACP's) recommendations.^3,4^ This article describes the programme's training model and the eight steps undertaken to operationalise the curriculum.

## Training model

The psychiatry residency training programme was designed to be a 3-year programme (Table 1). Historically, the training of West African medical graduates depends on independent learning and apprenticeship. Limited literature details how to implement the broad curricula mandated by accrediting bodies into the daily training structure, particularly in low-income countries with fewer personnel to dedicate time to educational implementation. Utilising the apprenticeship model in low-resource settings means that less time is needed to plan lectures and teach, thus allowing the allocation of resources to active clinical care, when specific teaching pearls can be bestowed. Apprenticeship requires active learning, corresponding to the graded experiential learning recommended in adult learning theory.^5^ Abas et al qualitatively analysed an apprenticeship model among Zimbabwean psychiatry residents, who noted that direct observation and immediate feedback in their high-volume clinical work increased clinical confidence and competence. However, the residents identified weaknesses in subspecialty knowledge due to training in general clinics without supplementary didactics to compensate for low-volume subspecialty cases.^6^ Another paper concluded that apprenticeship models can yield positive community health outcomes through active learning and post-training supervision.^7^

**Table 1:** Overview of the Liberian Psychiatry Residency Program

Clinical rotation	Specialty	Duration, months	Psychotherapy	Mental healthcare management
1	General adult psychiatry (includes aspects of rehabilitation and emergency psychiatry)	12	Psychotherapy and emergency psychiatry were covered throughout the programme	Mental healthcare management (clinical audit, monitoring and evaluation) was covered throughout the programme
2	Child and adolescent psychiatry (includes intellectual disability across the lifespan)	6		
3	Consultation-liaison psychiatry	3		
4	Neurology	3		
5	Geriatric/old age psychiatry	3		
6	Community, social and rehabilitation psychiatry	3		
7	Addiction psychiatry	3		
8	Forensic psychiatry	3		
	**Total**	**36**		

In contrast, the current trend in North American graduate medical training is to protect time from clinical service for structured seminars.^4,8^ Residents are expected to integrate the information experientially during clinical rotations. Although this model may better ensure coverage of less common clinical scenarios, residents’ engagement in didactics is variable, often reverts to passive learning and may be perceived to take lower priority than clinical work.^9^ With didactic time protected, residents may dedicate less time to self-directed learning owing to learning fatigue or a belief that content will be taught to them.^10^

In recognition of the comparative strengths and weaknesses of the West African apprenticeship and North American didactic models, the Liberia Psychiatry Residency Program used a hybrid model. The two full-time Liberia-based psychiatrists prioritised experiential learning while American partners focused on interactive didactic lectures in fundamental topics thought to vary least by culture, such as basic neuroscience. On occasions when US-centric clinical circumstances, diagnostic classifications or treatment approaches were presented, US faculty strove to acknowledge and highlight their cultural biases. The training team attempted to learn from the common pitfalls of cross-cultural collaborations.^11^

## Steps in curriculum development

### Step 1: knowledge assessment

The Liberia Psychiatry Residency Program started in July 2019 with three residents with diverse medical school experiences. The first step in programme development was to conduct assessments of the trainees’ knowledge, to determine initial teaching objectives. In addition to noting national entrance examination scores, faculty observed the new residents’ early psychiatric interviews and presentations to gauge their fundamental knowledge and skills. The programme prioritised providing greater neurological training to the first-year residents than is required by the WACP curriculum because Liberia had no trained neurologists, and the treatment of certain neurological cases, such as epilepsy, was managed by psychiatrists.

The knowledge assessments demonstrated wide variation in prior exposure to basic psychiatry among the three residents. All three had limited exposure and knowledge of neuroanatomy, neuroscience and the neurology physical examination. Although the residents generally understood the process of the psychiatric interview, knowledge about logically presenting the gathered information to a supervisor and using it to generate an assessment and plan was limited.

### Step 2: setting immediate and short-term priority topics

Using the results of the knowledge assessments, the faculty prioritised four areas: the psychiatric interview, case formulation, basic neuroscience and neurological examination. Following an iterative process, residents and faculty incorporated multiple cultural perspectives and preferences into an agreed methodology for teaching the psychiatric interview and formulation.

Faculty dedicated 6 months to a neuroscience series to strengthen the residents’ limited prior exposure to these topics. The neuroscience curriculum was adapted from existing curricula in the literature and included 12 didactic topics with an emphasis on clinical correlates (Box 1). The neurological examination was taught virtually via video conference and was reinforced in person through live demonstration, followed by observed evaluation and feedback. Residents participated in an annual observed neurological examination to ensure the continued development of their skills. Clinical neurology topics were presented via highly interactive lectures that were delivered by international faculty in disease-specific categories (Box 1).

Box 1 Neuroscience and neurology didactic topics for first-year psychiatry residents

**Neuroscience topics**

- Neuroscience and its relevance to psychiatry
- Basic neuroanatomy
- Synaptic transmission and neurotransmitters
- Somatic sensation and special senses
- Pyramidal and extrapyramidal system
- Emotion, language and memory
- Association cortices and executive function
- Arousal, sleep and sex
- Psychoneuroendocrinology and psychoneuroimmunology
- Neuroimaging
- Epigenetics and trauma
- Brain development across the lifespan

**Clinical neurology topics**

- Epilepsy
- Head injury
- Neurological examination
- Headaches and neurological emergencies
- Stroke
- Stroke in the young
- Dementia

### Step 3: establish faculty roles and meet regularly

Beyond the priority topics described above, it was critical to identify roles for individual faculty members in curriculum development and administration. These roles were not formalised until several months after the start of the residency in response to programme needs and faculty strengths. It is recommended that programmes replicating our model recognise the necessity of these faculty roles early. A US-based programme manager served as international liaison and administrative coordinator.^2^ The programme manager familiarised US faculty with the residency programme, arranged travel for US-based faculty, provided residents with, for example, access to an electronic database of journals and other educational materials, research assistance and so on and drafted grant proposals to promote programme longevity.

The physician faculty roles were divided into clinical and didactic components. As the only two psychiatrists physically in the country, the Liberia-based faculty oversaw ward- and clinic-based supervision of residents. One of these faculty members taught primarily through case supervision, while the other included formal didactics, often based on cases seen by the residents. The US-based faculty relied on Liberia-based colleagues for clarity on regional differences in patients’ access to healthcare, presentation and treatment to allow for cultural differences between the collaborating countries. US-based faculty taught via video teleconference on scheduled didactic days.

In addition to teaching weekly didactics, a US-based programme director focused on pedagogy and curriculum development to ensure synergy between the different cultures, countries and teaching faculty. The US-based associate programme director focused on basic neurological pedagogy and taught neurology didactics, focusing on diseases most prevalent at the training sites.

US-based and Liberia-based faculty met virtually twice monthly for administrative check-ins regarding logistics, curriculum, residents’ feedback and residents’ progress.

### Step 4: teach in parallel modules

Owing to differences in teaching style and physical location, the programme leaders allocated teaching in psychiatry and neurology to individual lecturers. The modules were taught in parallel so that residents could develop knowledge in multiple domains despite the limited number of faculty. The content from each module was then integrated and clinically applied with supervision by Liberia-based faculty. Programme leaders used this method to ensure educational continuity and avoid unnecessary redundancy.

### Step 5: introduce guest lecturers

Guest lecturers were recruited to teach various practice styles, expanding residents’ knowledge. Areas taught included, for example, selected topics in forensic psychiatry, old age psychiatry, research methods and statistics. In the inaugural year, most guest lecturers were members of the African diaspora, and many had clinical training experiences in their country of origin. Others had prior experience in the West African region, with clear career investment in continuing this. Before guest lecturers began their teaching of residents, programme leaders described the programme and encouraged lecturers to use an interactive style and inquisitive strategy if they were not personally familiar with clinical practice in Liberia. In areas where cultural differences were most likely to affect best practice and there were no local content experts (e.g. psychotherapy), Liberia-based faculty members were invited to co-present and offer local perspectives.

### Step 6: planning ahead

Since the residency programme was launched in the context of limited mental health infrastructure, programme leaders needed to identify opportunities to immerse the residents in a variety of psychiatric settings as required by accreditation guidelines. The three existing clinical settings were a mixed-sex in-patient unit, a women's stepdown unit and the local general hospital.^2^ Faculty are proactively expanding clinical opportunities through creative networking. For example, since there are no forensic and community psychiatry opportunities, faculty are developing relationships with the Ministry of Justice of Liberia, the local prison and rural clinics. Similarly, faculty have liaised with physicians in the general hospital to develop a consultation-liaison service that satisfies training requirements gradually.

### Step 7: in-training assessment

To assess residents’ knowledge and the quality of teaching, written and clinical skills examinations were administered in the middle and at the end of the first training year. Residents’ neurological examination skills were assessed using a structured rubric. They were also scored using the WACP template during a live psychiatric interview followed by a discussion of their clinical assessment. The written assessments comprised psychiatry and neurology multiple choice questions adapted for context by locally practising faculty. These assessments of clinical decision-making skills and treatment choices were based on recognised global best practice and aligned with the WACP standards rather than available treatments in the residents’ local context. In addition, a formative assessment by Liberia-based faculty was conducted.

### Step 8: obtain feedback and iterate

As the final step in implementing the first year of the Liberia Psychiatry Residency Program, the faculty collected verbal feedback along with anonymous written responses from the residents to understand what worked well or required modification. Based on feedback received, faculty are implementing changes for subsequent years. Most notably, residents requested earlier notice of their academic schedule and less redundancy in the programme, which will be accomplished through inventory and curation of the first-year experiences.

## Outcomes and reflections

Despite having only two psychiatrists physically based in Liberia, the residency programme expanded its faculty to include four core members and multiple guest lecturers, thereby increasing residents’ exposure to different clinical practices. The faculty covered psychotherapy, child psychiatry, basic acute and in-patient neurology, neuroscience, psychopharmacology, physiology and some psychiatric disease-specific modules in the first year, successfully implementing the hybrid model.

In addition to psychiatry residents, formal didactic sessions were also attended by medical students and physicians in family medicine and internal medicine. This increased acknowledgment of, and interest in, mental illness and created opportunities for collaboration.

There were multiple challenges to implementing the Liberia Psychiatry Residency Program. The most persistent challenges remain in identifying long-term funding and meeting accreditation requirements. On several occasions, the number of actively training residents was limited by challenging life circumstances, including family responsibilities, personal health issues and the SARS-CoV-2 pandemic, which delayed some rotations and postponed the start date of the second class of trainees by 2 months. An 8 h time difference between US-based and Liberia-based faculty complicated the logistics of scheduling academic opportunities and administrative meetings. Although programme leaders successfully scheduled meetings every 2–4 weeks, it was difficult for all parties to be consistently available. The physical presence of only two psychiatrists in Liberia resulted in multiple responsibilities and limited time to meet all the demands placed on them. For this reason, many administrative tasks were supported by US-based faculty, who had the advantage of protected time and could seek feedback and final approval from faculty in Liberia.

## Conclusion

With the successful completion of the Liberia Psychiatry Residency Program's inaugural year, residents gained significant knowledge and clinical skills quickly. This initiative demonstrates that mental health providers, even at advanced levels, can be further upskilled despite minimal infrastructure. By training academic psychiatrists, we intend to train the next generation of faculty educators who will, in turn, grow the training cohort and advance local mental health research within Liberia. The model described in this paper, with specific examples from the Liberian context, may help other new residency programmes in resource-constrained settings identify a pathway to successful curricular implementation, thereby increasing psychiatric capacity.

## Data Availability

Data are available on reasonable request.
